# Therapeutic Potential of Minor Cannabinoids in Dermatological Diseases—A Synthetic Review

**DOI:** 10.3390/molecules28166149

**Published:** 2023-08-20

**Authors:** Emilia Kwiecień, Dorota Kowalczuk

**Affiliations:** 1 Chair and Department of Medicinal Chemistry, Faculty of Pharmacy, Medical University of Lublin, Jaczewskiego 4, 20-090 Lublin, Poland; emilia.kwiecien@a-sense.pl; 2 A-Sense Sp. z o.o., ul. Moscickiego 1, 24-100 Pulawy, Poland

**Keywords:** minor cannabinoids, CBDV, THCV, CBDP, CBC, CBGA, dermatological diseases

## Abstract

Dermatological diseases pose a significant burden on the quality of life of individuals and can be challenging to treat effectively. In this aspect, cannabinoids are gaining increasing importance due to their therapeutic potential in various disease entities including skin diseases. In this synthetic review, we comprehensively analyzed the existing literature in the field of potential dermatological applications of a lesser-known subgroup of cannabinoids, the so-called minor cannabinoids, such as cannabidivarin (CBDV), cannabidiforol (CBDP), cannabichromene (CBC), tetrahydrocannabivarin (THCV), cannabigerolic acid (CBGA), cannabigerol (CBG), cannabielsoin (CBE), cannabimovone (CBM) or cannabinol (CBN), while drawing attention to their unique pharmacological properties. We systematically searched the available databases for relevant studies and analyzed the data to provide an overview of current thematic knowledge. We looked through the full-text, bibliographic and factographic databases, especially Scopus, Web of Science, PubMed, Polish Scientific Journals Database, and selected the most relevant papers. Our review highlights that minor cannabinoids exhibit diverse pharmacological activities, including anti-inflammatory, analgesic, antimicrobial, and anti-itch properties. Several studies have reported their efficacy in mitigating symptoms associated with dermatological diseases such as psoriasis, eczema, acne, and pruritus. Furthermore, minor cannabinoids have shown potential in regulating sebum production, a crucial factor in acne pathogenesis. The findings of this review suggest that minor cannabinoids hold therapeutic promise in the management of dermatological diseases. Further preclinical and clinical investigations are warranted to elucidate their mechanisms of action, determine optimal dosage regimens, and assess long-term safety profiles. Incorporating minor cannabinoids into dermatological therapies could potentially offer novel treatment options of patients and improve their overall well-being.

## 1. Introduction

Skin diseases such as acne, psoriasis, atopic dermatitis, and other dermatological conditions are prevalent and often cause not only physical discomfort but also emotional and social distress. Modern medicine offers numerous treatment options, but they do not always yield the desired outcomes. In the search for new, effective, and safe therapeutic methods, attention has turned to major cannabinoids (such as cannabidiol (CBD) and tetrahydrocannabinol (THC) that occur naturally in fiber hemp (*Cannabis sativa L.*) and Indian hemp (*Cannabis indica Lam.*) in larger quantities, as well as minor cannabinoids (such as cannabidivarin (CBDV), cannabidiforol (CBDP), cannabinol (CBN), cannabigerol (CBG), cannabichromene (CBC), tetrahydrocannabivarin (THCV), cannabielsoin (CBE), cannabimovone (CBM), cannabigerolic acid (CBGA), and other acid forms of cannabinoids), which occur naturally in very low concentrations.

In the presented study, our focus is on providing an overview of the available knowledge regarding minor cannabinoids and discussing their potential therapeutic applications in skin diseases. A review of the scientific literature allows the identification of the latest procedures, research, and findings concerning the efficacy and safety of cannabinoids. Furthermore, we discuss the mechanisms of action of these compounds, methods of extraction/synthesis, and their impact on pathological processes occurring in the skin. This publication aims to provide up-to-date information for medical professionals, scientists, and patients interested in the therapeutic use of cannabinoids in skin diseases.

## 2. Methods of Obtaining Cannabinoids

### 2.1. Conventional Extraction Methods

**Figure 1:**
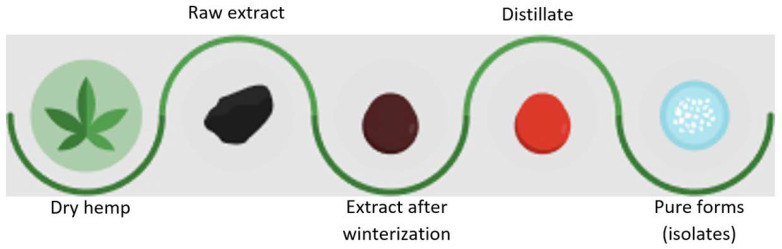
Scheme of hemp extract processing to crystalline forms of cannabinoids.

The oldest and likely most commonly used methods of obtaining cannabinoids are traditional methods of isolating them from hemp plants, represented by various extraction techniques such as solvent extraction, liquid–liquid extraction, and supercritical fluid extraction. Solvent-based extraction techniques (using solvents such as ethanol or hexane) utilize the solvent’s solvating properties. On the other hand, supercritical fluid extraction exploits both the solvating properties of the liquid and the penetrating properties of gases (such as supercritical carbon dioxide) to extract cannabinoids from plant material. Although solvent-based extraction methods are relatively simple and accessible, they have some drawbacks, including relatively low extraction efficiency, the risk of solvent residues in the final product after evaporation, and potential hazards associated with flammable solvents [1,2,3]. On the contrary, supercritical carbon dioxide extraction allows for obtaining solvent-free extracts, is more efficient, and enables the extraction of acid forms (e.g., cannabidiolic acid (CBDA), CBGA) from hemp material. The obtained hemp extracts then undergo further processing to eliminate undesirable plant components (e.g., waxes). After the cannabinoid separation step from the raw plant material, the fresh extract undergoes winterization to remove impurities such as waxes and chlorophyll. Subsequently, in the distillation process, cannabinoids are separated from other compounds like terpenes and flavonoids to obtain a concentrated product. Following distillation, a crystallization process is conducted by controlled cooling to precipitate pure cannabinoid crystals. Ultimately, cannabinoid crystals are dried and can take the form of a solid powder or crystals (e.g., CBD, CBG, CBDV, CBN, CBGA) (Figure 1). Some cannabinoids do not crystallize, and their purification is most commonly achieved using chromatographic column [4,5].

### 2.2. Chemical Synthesis

Chemical synthesis is a process that involves transforming available chemical precursors into desired cannabinoid molecules through chemical reactions such as condensation, acylation or reduction. Chemical synthesis offers a wide range of possibilities for structural modifications, which can lead to cannabinoids with increased activity, selectivity, or stability [6]. The condensation method is used to create cannabinoids through chemical reactions between precursors, forming the cannabinoid ring structure. The reduction method involves transforming cannabinoid precursors into active cannabinoids through electronation processes. The Friedel–Crafts method relies on electrophilic aromatic substitution reactions, where desired cannabinoids are formed through the reaction of aromatic compounds with electrophiles. All these methods require quality control, analysis, and specialized knowledge to ensure the purity and compliance of the obtained cannabinoids with specific standards [7,8,9].

In practice, the most commonly used compounds for synthesis are olivetol or its methyl/ethyl esters with varying carbon chain lengths on the olivetolic acid ring. For example, in the synthesis of CBDV, olivetolic acid (or its esters) with a three-carbon side chain is used, while in the synthesis of CBDP, it is used with a seven-carbon side chain [10,11].

### 2.3. Biotechnological Synthesis

The utilization of living organisms, such as bacteria or yeast, for the production of cannabinoids is another promising approach. Biotechnological methods rely on genetic engineering, which enables the introduction of genes encoding enzymes responsible for cannabinoid synthesis into the host organism. As a result, these organisms become factories for cannabinoid production, which can contribute to increased efficiency and process control [12,13,14]. This process involves several stages that can be divided into general categories: genetic modification of the host organism and the biosynthesis stages of the cannabinoid itself. Genetic modification of the host organism involves selecting a host organism which can be a bacterium such as *Escherichia coli* (*E. coli*) or yeast such as *Saccharomyces cerevisiae* (*S. cerevisiae*). An organism that is easily culturable and genetically manipulable is chosen. In the second step, the genes responsible for cannabinoid production need to be identified and isolated. In the case of CBDV, these are the genes that encode enzymes responsible for the conversion of chemical precursors into CBDV. The final stage is genetic modification. The genes responsible for cannabinoid synthesis are introduced into the genome of the host organism through genetic engineering techniques such as genetic transformation. This may involve inserting genes from other organisms or manipulating existing genes to achieve the desired effect. The precursor can be CBG, CBGA, or another compound that can be transformed into the specific cannabinoid. Enzymes encoded by the introduced genes catalyze the chemical reactions that convert the precursor into the cannabinoid, such as CBDV. The specific steps depend on the specific enzymes used in the biosynthesis process. After the completion of the biosynthesis process, for example, CBDV, the cannabinoid can be extracted from the host organism and undergo further purification and refinement steps to obtain a pure product. The biosynthesis method allows for controlled and efficient production of CBDV in larger quantities than possible with extraction from hemp plants, which can be significant for research, pharmaceutical, and industrial purposes [15,16].

### 2.4. Enzymatic Synthesis

Enzymatic synthesis is a process in which enzymes are used to transform chemical substrates into cannabinoids. Various enzymes, such as tetrahydrocannabinolic acid synthase (THCAS), cannabigerolic acid synthase (CBGAS), and cannabidiolic acid synthase (CBDAS), are involved in the enzymatic synthesis of cannabinoids. These enzymes catalyze chemical reactions that lead to the production of specific cannabinoids. In the context of the aforementioned processes, enzymatic synthesis can be more selective and specific, allowing for controlled production of specific cannabinoids [17,18].

## 3. Mechanisms of Action of Cannabinoids on the Skin and Their Impact on Pathological Processes Occurring in the Skin

Cannabinoids interact with the body through the endocannabinoid system, a central regulatory system responsible for maintaining the health and proper functioning of almost every organism. The endocannabinoid system consists of cannabinoid receptors CB1 and CB2, endogenous compounds, and metabolic enzymes, and its function is to maintain homeostasis in the body, including controlling inflammation and pain [19]. Phyto-cannabinoids are plant counterparts of human endocannabinoids, such as 2-arachidonoylglycerol and anandamide, and they mimic their actions by stimulating cannabinoid receptors CB1 and CB2. Increasing evidence suggests that endocannabinoid signaling plays a crucial role in regulating biological processes in the skin. Many skin functions, such as immune response, cell proliferation, differentiation, and survival, are at least partially regulated by the endocannabinoid system, and suppressing skin inflammation is one of its strongest functions [20]. The impact of cannabinoids on pathological processes occurring in the skin may be related to their anti-inflammatory, antibacterial, antioxidant, and immunomodulatory properties. Cannabinoids contribute to reducing skin inflammation by inhibiting the release of pro-inflammatory cytokines and inducing the secretion of anti-inflammatory cytokines. Furthermore, they inhibit the activity of enzymes involved in lipid production, which helps reduce sebum production and prevent acne formation. They also exhibit antibacterial effects, particularly against bacteria of the *Staphylococcus* genus, which are often responsible for skin infections, and *Cutibacterium acnes* (*C. acnes*), one of the causes of common acne [21,22]. Cannabinoids have antioxidant activity, which can help prevent skin damage caused by free radicals and protect against photoaging. The influence of cannabinoids on the skin’s immune system may result from regulating cytokine production and inhibiting the activity of immune cells. In the case of autoimmune skin diseases such as psoriasis or atopic dermatitis, inhibiting the immune response can lead to reduced inflammation and alleviation of symptoms. The mechanisms of action of cannabinoids in skin diseases are complex and require further research. However, existing results suggest that they may be effective and safe methods for treating skin diseases [23,24,25].

The skin, as the largest organ of the body, manifests a complex system of neuroendocrine interactions. Neurotransmitters, neuropeptides, hormones and signalling factors play a key role in the regulation of skin processes, interacting with dermal–vascular receptors and the endocannabinoid system [26,27,28]. An important aspect is the influence of cannabinoids, plant or synthetic compounds, which interact with cannabinoid receptors in the skin to regulate neuroendocrine processes. Cannabinoid receptors, mainly CB_1_ and CB_2_, present in skin cells and the nervous system, form the foundations of the interaction between the endocannabinoid system and skin neuroendocrinology [29,30,31]. In the context of neuroendocrinology, cannabinoids can influence hormone secretion by interacting with cell receptors or indirectly, by influencing stress responses. For example, stress can induce the activation of the endocannabinoid system, which consequently affects skin hormonal responses. Melanotropin is linked to the endocannabinoid system through its interaction with CB1 receptors present in the skin. This interaction may influence the regulation of melanin production and the modulation of the skin stress response, implying potential involvement of endocannabinoids in neuroendocrine processes related to skin pigmentation and adaptation. The skin expresses elements of the hypothalamic–pituitary–adrenal axis control, including POMC (pro-opiomelanocortin) and its derivatives. Receptor interactions indicate autocrine mechanisms of action. Cutaneous hormone production, including vitamin D3 and PTHrP (parathyroid hormone-related protein), modulated by stressors, influences stress responses. The skin’s neuroendocrine system communicates at both local and systemic levels, impacting vascular, immune, and pigmentary alterations. This implies that it functions to maintain skin integrity and systemic homeostasis. Skin aging leads to the loss of function and adaptive capabilities to stress, resulting from intricate biological processes influenced by genetics, environment, and pathology. Environmental factors such as UV radiation and pollutants expedite this process, disrupting cutaneous neuroendocrine systems. Topical application of neurohormonal substances, like melatonin, secosteroids and cannabinoids may mitigate skin aging effects through targeted interaction with receptors and enzymes [26,27,28,29].

## 4. Cannabinoids in Dermatological Disorders

### 4.1. Cannabinoids in Psoriasis

Psoriasis is a skin disease characterized by accelerated skin cell turnover and inflammation. Although the exact causes of this disease are not fully understood, the endocannabinoid system plays an important role in its development. Inhibiting excessive proliferation of keratinocytes is one way to alleviate psoriasis symptoms [32,33]. Cannabinoid receptors CB1 and CB2 influence the expression of pro-inflammatory cytokines, which are one of the triggering factors for psoriasis. The activation of CB2 receptors decreases the release of pro-inflammatory cytokines, while the activation of CB1 receptors increases their production [34,35]. Cannabinoids also affect receptors activated by peroxisome proliferator-activated receptors (PPAR) and transient receptor potential vanilloid 1 (TRPV1). In psoriasis, especially in the early stage of the disease, there is an increase in oxidative stress in granulocytes and serum [36]. Mitochondria are the main source of reactive oxygen species (ROS), and the increase in ROS is dependent on increased lymphocyte activity [37]. The antioxidant capacity of cannabinoids has been studied using various methods, such as the DPPH (2,2-diphenyl-1-picrylhydrazyl) assay, ABTS (2,2′-azino-bis(3-ethylbenzthiazoline-6-sulfonic acid)) assay, CUPRAC (CUPric Reducing Antioxidant Capacity) assay, and ORAC (Oxygen Radical Absorbance Capacity) assay. Depending on the method used, the antioxidant potential of cannabinoids varies, but there is a certain pattern observed in the research results. CBG, compared to CBD, exhibits twice the antioxidant activity [38], which is attributed to the terpene structure present in the CBG molecule, unlike the olivetol structure that is identical in both molecules. On the other hand, acidic forms of cannabinoids can form hydrogen bonds between the hydroxyl and carboxyl groups. In theory, such bonding would reduce the antioxidant activity. However, in practice, cannabinoid acids do not lose their antioxidant properties, likely due to various mechanisms such as neutralizing reactive oxygen species, inducing antioxidant enzymes, and modulating oxidative signaling pathways [39]. Additionally, CBD inhibits the activity of NADPH (nicotinamide adenine dinucleotide phosphate hydrogen) enzymes and xanthine oxidase in keratinocytes, which contribute to increased oxidative stress [13]. Studies have shown that CBD reduces the activity of TNF-α (tumor necrosis factor-alpha), lymphocyte proliferation, and protects cells from the effects of UV radiation, thereby reducing the inflammatory response at the site of action [13,40,41].

The anti-inflammatory effects, the inhibition of keratinocyte proliferation, and the impact of angiogenesis on the pathogenesis of psoriasis were described by Amir Hossein Norooznezhad and Fatemeh Norooznezhad. The researchers highlighted the potential of CBN, which significantly inhibits excessive keratinocyte proliferation [42]. Similar effects were found in earlier studies conducted by Jonathan D. Wilkinson and Elizabeth M. Williamson, demonstrating the potential of CBN. In these studies, a comparison of the inhibitory strength of keratinocyte proliferation was performed based on the dose of the cannabinoid used. The properties of Δ9-tetrahydrocannabinol (Δ9-THC), CBD, CBG, and CBN were compared after 72 h of administration. The determined IC₅₀ values, representing the concentration of the cannabinoid required to inhibit 50% of keratinocyte proliferation in vitro, ranged from 2 to 3 µM. Δ9-THC exhibited the lowest activity with an IC50 of 2.9 µM, while CBD showed the highest activity with an IC_50_ of 2 µM. CBN had a slightly lower IC50 of 2.1µM, and CBG had an IC_50_ of 2.3 µM [43].

Research has shown that cannabichromene (CBC) inhibits the proliferation of keratinocytes, which are overproduced in the case of psoriasis. CBC acts by inhibiting the Wnt/β-catenin signaling pathway responsible for controlling the proliferation of skin cells. Inhibiting this pathway may help alleviate symptoms of psoriasis such as redness and skin flaking [34,35]. Furthermore, CBC also exhibits anti-inflammatory activity, which can help alleviate inflammation in the skin. CBC inhibits the activity of pro-inflammatory cytokines and other inflammatory mediators, contributing to the reduction in inflammation and alleviation of psoriasis symptoms. However, further research on the efficacy of CBC and its mechanisms of action is needed to better understand the therapeutic potential of this cannabinoid in psoriasis [44,45].

Not only natural cannabinoids are the subject of scientific research. Researchers, including Judith A. Stebulis et al., investigated ajulemic acid, a synthetic cannabinoid acid, for its anti-inflammatory properties. Ajulemic acid increases the synthesis of 15d-PGJ2, a prostaglandin involved in dampening inflammation. Its mechanism of action involves the suppression of cyclooxygenase-2 (COX-2), which is not the most ideal alternative when it comes to anti-inflammatory preparations. Therefore, further research and the development of therapeutic alternatives are needed in this direction of investigation [46].

### 4.2. Cannabinoids in Acne

Acne is one of the most common skin disorders that affects millions of people worldwide. By nature, acne is a chronic condition characterized by skin lesions occurring on the basis of seborrhea. Noticeable features include excessive sebum production, enlarged pores, hyperkeratinization, and proliferation of *C. acnes* bacteria within the pilosebaceous unit. The pathogenesis of acne is complex and involves multiple factors, including increased levels of androgens, disrupted skin microbiome, as well as genetic and environmental factors. Depending on the visible skin lesions such as blackheads, papules, nodules, cysts, pustules, or scars, and the severity of these manifestations, various types of acne can be distinguished. In addition to the visual aspects, acne often has a significant impact on one’s psychological well-being, leading to low self-esteem and causing serious emotional and psychological problems. In this regard, neuropathic acne is also recognized, which can be caused by self-inflicted skin damage and picking at the existing lesions. Although acne commonly occurs in teenagers, it can also affect adults of different ages. Despite acne being a prevalent skin condition, each case is unique and requires an individual approach from a dermatologist [47].

Preliminary clinical studies indicate that topical cannabinoids such as CBD may be beneficial in the treatment of acne and skin rejuvenation [48]. Research conducted by Attila Olah et al. [49] suggests that CBG and CBGV may have potential in treating dry skin conditions, while CBC, CBDV, and especially THCV may be highly effective anti-acne agents. Other cannabinoid derivatives that demonstrate promising anti-acne properties include synthetic compounds such as WIN-55,212-2 and the previously mentioned ajulemic acid. WIN-55,212-2 is a synthetic cannabinoid and agonist of the CB1 and CB2 cannabinoid receptors. Studies have shown that WIN-55,212-2 can reduce sebum production and skin inflammation. Ajulemic acid, on the other hand, is a synthetic cannabinoid derivative and agonist of the PPAR-gamma receptor. Similar to WIN-55,212-2, ajulemic acid can reduce sebum production and skin inflammation, making it a potential anti-acne medication [47,50,51].

THCV is a cannabinoid that shows many promising properties in combating acne. According to conducted studies, THCV may help regulate sebum production, which is one of the main factors contributing to acne development. Additionally, THCV exhibits anti-inflammatory and antibacterial properties that can help alleviate inflammation and combat the bacteria responsible for acne development. Animal testing results suggest that THCV may have potential in treating metabolic and neurological disorders at doses ranging from 1 to 10 mg/kg of body weight. However, there is insufficient research indicating the optimal THCV doses for acne treatment in humans [52,53,54].

### 4.3. Cannabinoids in Atopic Dermatitis (AD)

AD is an inflammatory skin disease characterized by dry and itchy skin, as well as inflammatory and cutaneous changes. The pathogenesis of AD is complex and involves multiple factors, including genetics, immune system response to allergens, skin condition, and environment. Genetics play a crucial role in the pathogenesis of AD. Studies have shown that mutations in genes related to the skin barrier, such as the filaggrin gene, are associated with a higher risk of developing AD. These mutations lead to a weakened skin barrier, increasing the skin’s sensitivity to external factors such as allergens and pollutants [55]. The immune system’s response to allergens is also an important factor in the pathogenesis of AD. Patients with AD tend to react to various allergens, such as plant pollen, dust, animal dander, and mites. In response to these allergens, the immune system releases cytokines and other chemical substances, leading to skin inflammation. The skin condition also plays a significant role in the pathogenesis of AD. Individuals with AD have a tendency to have dry and irritated skin, which weakens the skin barrier and increases sensitivity to allergens and irritants. Moreover, excessive sebum production can lead to clogged skin pores and the formation of acne, worsening the skin condition in AD patients. There are studies suggesting that certain cannabinoids may exhibit therapeutic effects in the treatment of AD [55,56].

CBG is one of the so-called minor cannabinoids, but it plays an important role in the therapy of AD. CBG exhibits many beneficial properties, such as anti-inflammatory, antioxidant, antibacterial, and antifungal effects. These actions make CBG a good candidate for AD therapy. One of the mechanisms of CBG action in AD is its ability to inhibit the activity of enzymes responsible for the synthesis of fatty acids, such as arachidonic acid. Arachidonic acid is an important mediator of inflammation in the skin, so inhibiting its production can help reduce inflammation in AD [57,58]. Due to its antibacterial and antifungal activity, CBG may be particularly important for patients with AD, as individuals with AD are prone to skin infections, especially bacterial and fungal infections. CBG can also be an effective tool in preventing these infections and improving skin health. In animal studies, CBG has shown positive effects in alleviating skin inflammation, including AD. In one study on mice with AD, CBG administration effectively reduced inflammation and water loss through the skin. These results suggest that CBG may be a promising therapeutic agent for patients with AD [59,60].

CBDV is a compound that is present in small amounts in the hemp plant. CBDV exhibits many beneficial properties, including anti-inflammatory activity. One of the mechanisms of CBDV action in AD is its ability to inhibit the activity of pro-inflammatory cytokines, such as interleukin 6 and 17A. These cytokines play a crucial role in the inflammatory process in AD, and inhibiting their activity can help reduce inflammation and alleviate symptoms of the disease [61,62]. In animal studies, CBDV has shown effectiveness in alleviating symptoms of the disease, such as itching and skin redness [63].

### 4.4. Cannabinoids in Allergic Contact Dermatitis (ACD)

Research suggests that cannabinoids may have potential applications in the treatment of ACD. ACD is an inflammatory skin condition that can be triggered by contact with allergens such as nickel or latex. Animal studies have shown that cannabinoids, including CBD and THC, may help alleviate symptoms of ACD by reducing inflammation and skin itching [64,65]. Cannabinoids may act through the modulation of the endocannabinoid system, which plays an important role in regulating inflammatory processes in the body. However, the introduction of cannabinoids into ACD therapy requires further clinical research to determine their effectiveness and safety in patients with this condition [66].

### 4.5. Cannabinoids with Potential Anti-Inflammatory Properties

CBE exhibits anti-inflammatory effects by decreasing the production of nitric oxide (NO) and the pro-inflammatory cytokine interleukin-6 (IL-6) while increasing the production of the anti-inflammatory cytokine IL-10 and anti-inflammatory markers such as the human ARG1 gene which encodes the protein arginase (Arg-1) [67].

CBM is a substance that exhibits various anti-inflammatory properties. It is a full agonist of the PPARγ (peroxisome proliferator-activated receptor gamma) receptor, meaning it activates it to its full extent. PPARγ plays a significant role in regulating inflammatory processes, so the activation of this receptor by CBM may contribute to reducing inflammation in the body. It is also a weak agonist of the TRPV1 (transient receptor potential vanilloid-1) and TRPA1 (transient receptor potential ankyrin 1) receptors. These receptors are involved in transmitting pain signals and regulating inflammatory states. Their activation can lead to pain reduction and alleviation of inflammation. CBM shows low affinity for CB1 and CB2 receptors. This means that CBM does not primarily act through these receptors, which may be important in avoiding undesirable effects associated with their activation, such as psychoactive effects associated with CB1 [68]. Table 1 summarizes the potential mechanisms of action of cannabinoids on the skin.

## 5. Limitations

The use of cannabinoids for therapeutic purposes encounters significant scientific limitations. Despite potential benefits demonstrated in in vitro and animal studies, compelling clinical evidence of their efficacy and safety in humans is lacking. Diverse organismal responses, potential adverse effects, lack of dose standardization, and the possibility of interactions with other medications pose challenges. Additionally, the impact on the nervous system, issues pertaining to product quality and regulation, as well as ethical and legal aspects, including those concerning legality, require comprehensive consideration. Therefore, despite the promising therapeutic prospects, the utilization of cannabinoids, especially the minor cannabinoids, necessitates further research, regulations, and a balanced approach to ensure benefits while minimizing potential health and societal risks [69,70].

## 6. Summary and Conclusions

In recent years, there have been numerous studies on the impact of cannabinoids on skin diseases such as acne, psoriasis, atopic dermatitis (AD), and allergic contact dermatitis (ACD). This review provides evidence that minor cannabinoids may also have therapeutic potential in alleviating dermatological conditions. CBDV, CBC, CBDP, and CBN have been identified as having therapeutic potential. CBDV, with its anti-inflammatory properties, can be used to alleviate skin symptoms such as itching and swelling in the treatment of AD. Furthermore, there is evidence that CBDV, due to its anti-inflammatory and antioxidant properties, can have a healing effect on acne lesions. Other recently discovered cannabinoids such as CBM and CBE have also demonstrated anti-inflammatory potential. They represent a novel alternative for conducting scientific research regarding specific disease conditions. Similarly, CBC, with its anti-inflammatory and antioxidant effects, may have a beneficial impact on the treatment of acne, psoriasis, and AD. CBDP, exhibiting anti-inflammatory and analgesic properties comparable to CBD and CBG, may have a positive influence on the treatment of acne and psoriasis. There is also evidence of the positive effects of CBN in the therapy of AD and ACD, likely due to its analgesic and calming properties. In summary, this work aimed to provide readers with an overview of cannabinoids and their actions that have a beneficial impact on the treatment of dermatological conditions. However, further research is needed to confirm their effectiveness and safety.

## Figures and Tables

**Table 1:** Summary of the potential effects of minor cannabinoids in various dermatological diseases.

Dermatological Diseases	Cannabinoid	Potential Mechanism of Action	Literature
Psoriasis	CBN CBC Ajulemic acid	Inhibition of excessive keratinocyte divisions; inhibition of the proliferation of keratinocytes; inhibition of the signaling of the Wnt/β-catenin pathway; inhibition of the activity of pro-inflammatory cytokines and other inflammatory mediators; increasing the synthesis of 15d-PGJ2—prostaglandins; the mechanism of action is the suppression of cyclooxygenase-2 (COX-2)	[13,40,41]
Acne	CBDV CBC THCV WIN-55,212-2 Ajulemic acid	Reduction in the production of sebum; inhibition of the growth of bacteria *C. acnes*; regulation of the secretion of sebum; anti-inflammatory and antibacterial properties	[47,48,49,50,51]
Atopic Dermatitis (AD)	CBG CBDV	The ability to inhibit the action of enzymes responsible for the synthesis of fatty acids, such as arachidonic acid, antibacterial and antifungal activity; the ability to inhibit the activity of pro-inflammatory cytokines such as interleukin 6 and 17A	[57,58,59,60,61,62]
Allergic Contact Dermatitis (ACZ)	CBD THC	Relieving the symptoms of ACZ by reducing inflammation and itching of the skin	[64,65,66]
Anti-inflamatory (requires further detailed research)	CBM CBE	Inhibition of pro-inflammatory biomarkers	[67,68]

## Data Availability

Not applicable.
